# Revisiting Folk Moral Realism

**DOI:** 10.1007/s13164-016-0300-9

**Published:** 2016-03-01

**Authors:** Thomas Pölzler

**Affiliations:** Department of Philosophy, University of Graz, Attemsgasse 25/II, 8010 Graz, Austria

## Abstract

Moral realists believe that there are objective moral truths. According to one of the most prominent arguments in favour of this view, ordinary people experience morality as realist-seeming, and we have therefore prima facie reason to believe that realism is true. Some proponents of this argument have claimed that the hypothesis that ordinary people experience morality as realist-seeming is supported by psychological research on folk metaethics. While most recent research has been thought to contradict this claim, four prominent earlier studies (by Goodwin and Darley, Wainryb et al., Nichols, and Nichols and Folds-Bennett) indeed seem to suggest a tendency towards realism. My aim in this paper is to provide a detailed internal critique of these four studies. I argue that, once interpreted properly, all of them turn out in line with recent research. They suggest that most ordinary people experience morality as “pluralist-” rather than realist-seeming, i.e., that ordinary people have the intuition that realism is true with regard to some moral issues, but variants of anti-realism are true with regard to others. This result means that moral realism may be less well justified than commonly assumed.

Moral realists believe that there are objective moral truths. One of the most prominent arguments in favor of this view starts from a hypothesis about ordinary people’s moral experience. Ordinary people experience morality as “realist-seeming”, proponents of the argument claim. In judging a thing right, wrong, good, bad, etc. it seems to us as if we state objective moral truths (e.g., Dancy 1986, p. 172; Huemer 2005). Moreover, the assumption that there are such truths underlies much of our moral practice (e.g., Brink 1989, pp. 24, 36), and we also have the intuition or belief that there are such truths (e.g., Devitt 2002, p. 7; Sayre-McCord 2009).^1^

That humans experience things in a certain way of course does not prove that things actually are that way. We all know how appearances can be deceiving. Various realists have argued, however, that the hypothesis that ordinary people experience morality as realist-seeming (henceforth simply the “experiential hypothesis”) at least provides strong prima facie reason to believe that realism is true (e.g., Brink 1989, pp. 24, 36; Dancy 1986, p. 172; Huemer 2005, p. 115; Sayre-McCord 2009; Devitt 2002, p. 7). Jonathan Dancy, for example, writes:

[W]e take moral value to be part of the fabric of the world; taking our experience at face value, we judge it to be the experience of the moral properties of actions and agents in the world. And […] we should take it in the absence of contrary considerations that actions and agents do have the sorts of moral properties we experience in them. This is an argument about the nature of moral experience, which moves from that nature to the probable nature of the world. (Dancy 1986, p. 172)

Anti-realists have sometimes criticized arguments from moral experience by casting doubt on their inductive validity (e.g., Joyce 2009b; Loeb 2007b).^2^ This article, in contrast, rather focuses on the truth of the experiential hypothesis. Recently, some proponents of this hypothesis have claimed that it is supported by psychological research on how ordinary people think about the philosophical foundations of morality. While most recent research has been thought to contradict this claim, four prominent earlier studies indeed seem to suggest a tendency towards realism: studies by Goodwin and Darley (2008); by Wainryb et al. (2004); by Nichols (2004); and by Nichols and Folds-Bennett (2003). My aim in this article is to show that the experiential hypothesis is not even supported by these early studies on folk moral realism.

One way of showing that the above studies fail to support the experiential hypothesis would be to object against psychological research on folk moral realism or its relevance for the assessment of this hypothesis in general. For example, it may be claimed that ordinary people’s intuitions about the existence of objective moral truths are so vague that they cannot be appropriately ascribed to variants of realism or anti-realism as discussed by metaethicists (Sinclair 2012, p. 168); or that research on folk moral realism addresses aspects of moral experience that the experiential hypothesis is not even supposed to apply to (see Brink 1989, p. 25 for an understanding of the experiential hypothesis that may be appealed to in this regard). Elsewhere (Pölzler 2014: 77–81) I argued that objections of this kind are rather unconvincing. My following criticism of the above studies will therefore be exclusively internal.

In Section 1 I will explain what proponents of the experiential hypothesis mean by “moral realism”. In Section 2 I will introduce psychological research on folk moral realism. In Sections 3 to 7, finally, I will provide a detailed analysis of the above mentioned studies and their implications for the assessment of the experiential hypothesis. I will argue that, once interpreted properly, all four early studies on folk metaethics turn out in line with more recent research. They suggest that most ordinary people experience morality as “pluralist-” rather than realist-seeming, i.e., that ordinary people have the intuition that realism is true with regard to some moral issues, but variants of anti-realism are true with regard to others.

## Moral Realism

**Fig. 1:**
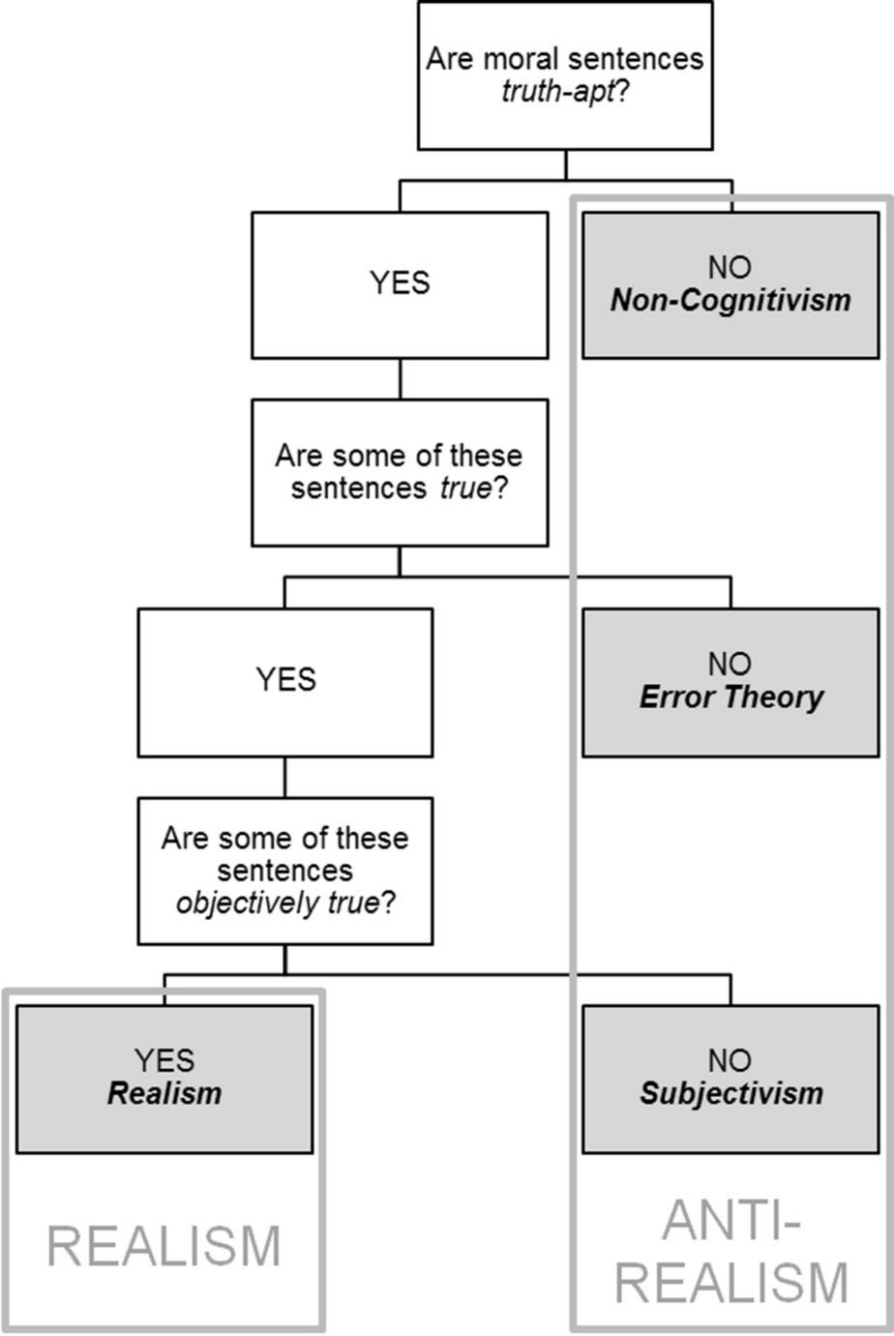
Variants of realism and anti-realism according to Huemer 2005, pp. 4–7

Just as metaethicists more generally, proponents of the experiential hypothesis have defined realism and anti-realism in various different ways. In this article I assume a definition proposed by Michael Huemer (2005), and in identical or very similar forms endorsed by many other metaethicists as well (see, e.g., Brink 1989; Joyce 2007a; Miller 2014, 2009).^3^ On this definition moral realism and anti-realism are about the existence of objective moral truths. Are moral sentences truth-apt? If yes, are some of these sentences actually true? And if yes, are these true sentences objectively true? While anti-realists deny at least one of these questions, realists affirm all of them (Huemer 2005, p. 4; see also Fig. 1 below).^4^

In order for the above definition of realism and anti-realism to be helpful one needs to have some idea of how to understand “truth” and “objectivity”. The sense of moral truth that is at issue in the realism/anti-realism debate is a robust one, i.e., for a moral sentence to be (objectively) true is supposed to mean that it correctly represents the (objective) moral facts (Huemer 2005, pp. 38–44). By “objectivity” realists and anti-realists typically mean observer- (or mind- or subject-) independence. On the specific conception of objectivity *qua* observer-independence assumed here (Huemer 2005, pp. 2–4), the relevant form of independence is a conceptual one (specifying what it means for a thing to have a certain property)^5^; the relevant kind of observers are any observers (human and non-human, actual and hypothetical); and the relevant kinds of mental states of these observers are any mental states that these observers have towards the thing at issue (beliefs, hopes, intentions, etc.). Thus, moral properties qualify as objective if and only if we can explain what it means for a thing to have these properties without referring to any mental state that any observer has towards that thing.

F-ness is subjective = Whether something is F constitutively depends at least in part on the psychological attitude or response that observers have or would have towards that thing. I define an ‘objective’ feature as one that is not subjective. (Huemer 2005, p. 2)

The existence of objective moral truths in the sense explained above can be affirmed and denied in various ways. For our purposes we need not bother with differences within realism (such as between the naturalism of Brink 1989 and the non-naturalism of Huemer 2005). It is rather important to be able to distinguish between three main variants of anti-realism: non-cognitivism, error theory and subjectivism.

According to non-cognitivism, the reason for there not being objectively true moral sentences is that moral sentences are not even truth-apt.^6^ In uttering such sentences people do not purport to represent facts, but rather only express non-cognitive mental states such as feelings of approval or disapproval, intentions or sentiments. A. J. Ayer, for example, famously claimed that to say “You acted wrongly in stealing that money,” is akin to saying “Stealing money is wrong” in “a peculiar tone of horror,” or to saying “Stealing Money: Boo!” (1952, p. 107; see also, e.g., Gibbard 1990; Blackburn 2000).

Error theorists are cognitivists, i.e., they believe that moral sentences are truth-apt. In contrast to realists, however, they hold that the facts that these sentences purport to refer to actually do not exist. This leads them to claim that all moral sentences are false — just as, for example, atheists believe that all theistic sentences are false, or most reasonable persons believe that all astrological sentences are false (see, e.g., Mackie 2011; Joyce 2001, 2007a, 2013; Lillehammer 2004; Pigden 2007).^7^

Subjectivists, finally, hold both that moral sentences are truth-apt and that some of these sentences are true. Where they depart from realism is with regard to the question of what makes true moral sentences true. While realists believe that these sentences are made true by objective facts, subjectivists believe that the relevant facts are subjective. Depending on the particular kind of observer-dependence that it attributes to moral facts, subjectivism can be held in many different variants. The four psychological studies on folk metaethics that will be considered below (partly unintentionally) mainly address individual subjectivism, cultural relativism and response dependence theory.

*Individual subjectivists* hold that a thing is right, wrong, good, bad, etc. if and only if the person who judges it in that way believes that it is right, wrong, good, bad, etc. *Cultural relativists* maintain that a thing is right, wrong, good, bad, etc. if and only if the culture in which the judgement is made predominantly judges the thing right, wrong, good, bad, etc. (e.g., Harman 1996). And according to *response dependence theorists*, the moral properties of things are determined by how observers respond to that thing under certain circumstances; for example, by whether humans under normal conditions respond to the thing by having certain emotions (Hume 1978; Prinz 2006, 2007), or by whether ideal observers would approve of it (Firth 1952).^8^

## Psychological Studies on Folk Moral Realism

Empirical psychology has long neglected metaethical intuitions.^9^ In the last 15 years, however, interest in them literally exploded (e.g., Beebe 2014; Beebe and Sackris forthcoming; Cova and Ravat 2008; Goodwin and Darley 2008, 2010, 2012; Nichols 2004; Nichols and Folds-Bennett 2003; Quintelier and Fessler 2012; Quintelier et al. 2013; Sarkissian et al. 2011; Wainryb et al. 2004; Wright et al. 2013, 2014; Young and Durwin 2013). So far most studies on folk metaethics have addressed the prevalence, causes or consequences of what researchers referred to as moral “objectivism” versus “subjectivism” or “relativism” (e.g., Goodwin and Darley 2008, p. 1341; Nichols and Folds-Bennett 2003, p. B23). These studies may therefore seem irrelevant to assessing the experiential hypothesis. However, as the labels “objectivism”, “subjectivism” and “relativism” have been used in these studies, they are largely or even fully equivalent to (variants of) realism and anti-realism as explained in Section 1 above.

By “objectivism” researchers on folk metaethics have typically meant what is here called “realism”, i.e., the view that moral truths are objective. According to Geoffrey Goodwin and John Darley, for example, objectivists claim that moral beliefs or standards “derive their truth (or warrant) independently of human minds (i.e., objectively)” (2008, p. 1341). Shaun Nichols and Trisha Folds-Bennett similarly define objectivism as the view that “(i) true moral judgments are nonrelativistically true and (ii) some moral judgments are true” (2003, p. B24).^10^ Moreover, when researchers have declared to explore folk “subjectivism” or “relativism” they have typically been concerned with what was labelled “subjectivism” above, i.e., with the view that moral truths depend on the mental states of observers; or, as Goodwin and Darley put it, the view that these truths are “entirely mind-dependent or subjective” (2008, p. 1341; see also, e.g., Nichols 2004, p. 7).

Given these taxonomic similarities it is no wonder that discussants of the experiential hypothesis have recently begun to develop interest in research on folk objectivism versus subjectivism/relativism (see, e.g., Joyce 2007c; Sinclair 2012). Research of this kind has in particular been claimed to *support* the experiential hypothesis. Richard Joyce, for example, writes^11^:

Research reveals that “common sense morality” does include certain claims to objectivity. […] moral prescriptions and values are experienced as “objective” in the sense that they don’t seem to depend on us, or on any authoritative figure. (Joyce 2007c, pp. 129–130)

Is this verdict warranted? Most recent studies clearly contradict Joyce’s assessment. As will be explained below (Section 7), they suggest a far more complex picture, with metaethical intuitions varying interpersonally or intrapersonally. Around the time of the publication of Joyce’s above quoted book, however, the claim that the available psychological evidence supports a tendency towards realism may well have been regarded as justified by many scholars in the field. For various early studies on folk moral realism really did report such a tendency, or have been claimed to show such a tendency by discussants. Most prominently, this holds for studies by Goodwin and Darley (2008), by Wainryb et al. (2004), by Nichols (2004); and by Nichols and Folds-Bennett (2003).^12^ Goodwin and Darley, for example, sum up the results of their study as follows:

Individuals seem to identify a strong objective component to their core ethical beliefs [...]. Arguably, many of our participants viewed their ethical beliefs as true in a mind-independent way. (Goodwin and Darley 2008, p. 1359)

Nichols and Folds-Bennett draw an analogous conclusion about children’s metaethical intuitions:

The findings of both experiments support the claim that children do not regard moral properties as response dependent. […] Together with previous findings […] these results suggest that children are indeed moral objectivists. (Nichols and Folds-Bennett 2003, p. B30)

In what follows I will argue that contrary to such interpretations, not even the above four early studies on folk moral realism provide any support for the experiential hypothesis. First, I will show that once interpreted properly, these studies are in line with more recent research. They too suggest that ordinary people’s intuitions about the existence of objective moral truths vary strongly (Sections 2 to 6).^13^ Then I will argue that this finding is mainly due to subjects’ intuitions varying intrapersonally, and that just as interpersonal variation, this intrapersonal variation is incompatible with the experiential hypothesis as well (Section 7). Note that throughout this discussion I will substitute researchers’ usage of the terms “objectivism” and “relativism” by their equivalents as defined above, i.e., by “realism” and “subjectivism”.

## Research Isolating Non-Cognitivism

The first early study on folk metaethics that I will analyze is Geoffrey Goodwin and John Darley’s groundbreaking 2008 study, in particular the first stage of the first and the first stage of the second experiment of this study. In both of these experiments subjects were presented a number of moral sentences, for example, “Anonymously donating a significant proportion of one’s income to charity is a morally good action,” or “Consciously discriminating against someone on the basis of race is morally wrong” (2008, pp. 1361–1362). For each of these sentences they were then asked a question that was supposed to reveal whether they tended towards realism or subjectivism (see Goodwin and Darley 2008, p. 1341).

In the first experiment the question that was thought to bring out subjects’ metaethical intuitions was whether they considered the given moral sentence “true”, “false”, or “an opinion or attitude”:

How would you regard the previous statement? Circle the number. (1) True statement. (2) False statement. (3) An opinion or attitude. (Goodwin and Darley 2008, p. 1344)

As Goodwin and Darley interpreted subjects’ responses, “true” and “false” responses indicated intuitions in favor of objectivism, and “opinion or attitude” responses intuitions in favor of subjectivism (see 2008, p. 1345):

1. “true” = Realism
2. “false” = Realism
3. “an opinion or attitude” = Subjectivism

In their second experiment Goodwin and Darley asked subjects whether they thought there was a “correct answer” regarding the truth of the moral sentences they were presented with:

According to you, can there be a correct answer as to whether this statement is true? (Goodwin and Darley 2008, p. 1351)

Subjects could respond by marking either “yes” or “no”. On Goodwin and Darley’s interpretation, “yes” responses indicated realist intuitions, “no” responses indicated subjectivist intuitions (see 2008, p. 1352):

1. “yes” = Realism
2. “no” = Subjectivism

Goodwin and Darley’s methodology has been subject to extensive criticism. First, it has been objected that as their question in Experiment 1 required subjects to make first-order rather than only second-order moral judgements, this question may have been misread as epistemic. Subjects may have chosen “true” or “false” if they were (strongly) convinced of the presented actions’ rightness/wrongness, and “opinion or attitude” if they were rather uncertain about it (Beebe and Sackris forthcoming, p. 5; Sinnott-Armstrong 2009, p. 244). Second, one may also worry that some “opinion or attitude” and “there is no correct answer as to whether this statement is true” responses are attributable to subjects regarding the relevant sentences as non-moral (Wright et al. 2013, p. 338). More recent research suggests that neither of these alternative explanations may be particularly significant, though (Goodwin and Darley 2010, p. 180; Wright et al. 2013, pp. 341–342, 344). In what follows I will therefore focus on what I take to be the above experiments’ main problem, namely their inadequate metaethical interpretation of subjects’ responses.

Consider experiment 1. Goodwin and Darley assume that those who answer that a given moral sentence is (R1) “true” or (R2) “false” tend towards realism, and those who answer that the sentence is (R3) an “opinion or attitude” tend towards subjectivism. But both of these categorizations are inadequate. To begin with, R1 and R2 are not only consistent with realism, but also with all other variants of cognitivism, i.e., with subjectivism and error theory. Subjectivists believe that moral sentences are true or false depending on whether they correctly represent the subjective moral facts. Error theorists believe that all moral sentences are false. Furthermore, R3 only appeals to non-cognitivists, for only they believe that moral sentences cannot be assessed in terms of truth or falsity at all. By contrasting subjects who opted for R1 and R2 with those who opted for R3, Goodwin and Darley thus did not measure the prevalence of realism versus subjectivism, but rather of cognitivism versus non-cognitivism:

1. “true” = Cognitivism (Realism or Subjectivism)
2. “false” = Cognitivism (Realism, Subjectivism or Error Theory)
3. “an opinion or attitude” = Non-Cognitivism

Similar considerations apply to Goodwin and Darley’s second experiment as well. The response that there is a correct answer as to whether a given moral sentence is true (R1) is not only entailed by realism, but by all variants of cognitivism. Subjectivists believe that the correct answer to the question of whether some moral sentence is true can be “yes” (if the sentence correctly represents the subjective facts) or “no” (if the sentence does not correctly represent these facts). Error theorists believe that the correct answer is always “no”, i.e., that the sentence is not true. Moreover, the response that there is *no* correct answer about whether a given moral sentence is true (R2) should only appeal to non-cognitivists, for only according to them it does not make any sense to ascribe truth or falsity to such sentences at all. Goodwin and Darley’s second experiment thus again measures the proportion of cognitivists versus non-cognitivists:

1. “yes” = Cognitivism (Realism, Subjectivism or Error Theory)
2. “no” = Non-Cognitivism

**Fig. 2:**
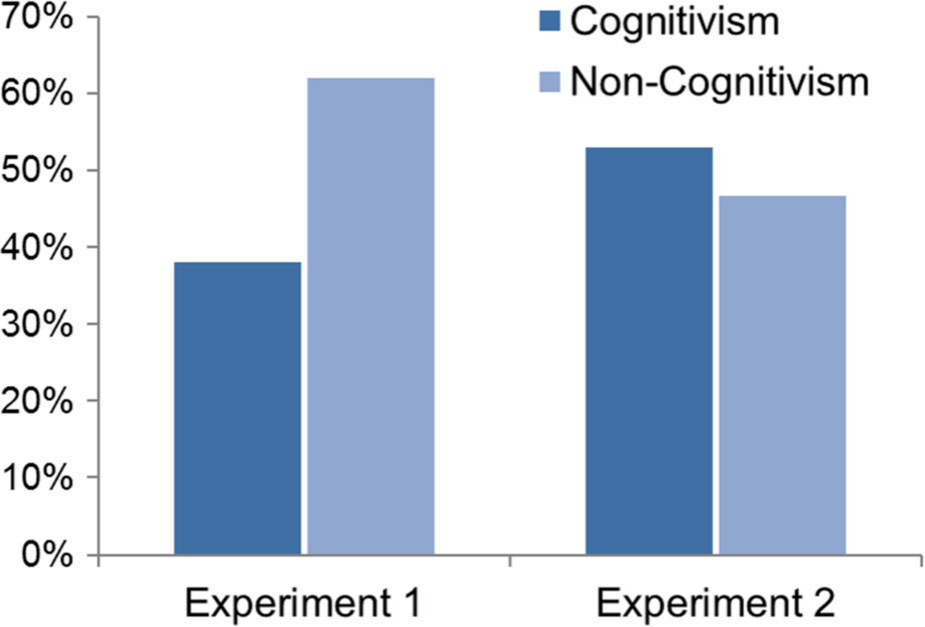
Distribution of responses in the first stage of Goodwin and Darley’s 2008 experiments (see pp. 1347, 1351) as reinterpreted above

The results of Goodwin and Darley’s experiments have often been claimed to suggest a tendency towards realism. Reinterpreted according to my above suggestions, however, this clearly is not the case. As many as 62 % of the responses of Experiment 1 and 47 % of the responses of Experiment 2 — in total more than half of the responses — belonged to the non-cognitivist options R3 (Experiment 1) and R2 (Experiment 2) (see 2008, pp. 1347, 1351; see Fig. 2). And the prevalence of intuitions in favor of anti-realism in general was likely even considerably higher than that. After all, subjects’ cognitivist responses can reflect anti-realist (in particular, error theoretic or subjectivist) commitments rather than realist ones as well.

## Research Isolating Individual Subjectivism

The next early experiments on folk metaethics that I will turn to are the second stages of Experiment 1 and 2 of Goodwin and Darley’s above 2008 study as well as an experiment with children between the ages of five and nine conducted by Wainryb et al. (2004). Subjects in both studies were confronted with one or more cases of moral disagreement. For example, they were told that another subject of the study had denied a moral sentence that they themselves affirmed (Goodwin and Darley 2008, p. 1362), or that two children disagreed about whether it is okay to hit and kick other children (Wainryb et al., p. 688). The principal question of the experiments was how subjects interpreted this disagreement (Goodwin and Darley 2008, p. 1363; Wainryb et al. 2004, p. 691). Goodwin and Darley, for example, wrote:

You circled __ on the scale (1–6) which means that you strongly **agreed/disagreed** with this statement. A person who we tested, strongly **agrees/disagrees** with this statement, which means that he or she sharply disagrees with you. What would you conclude about this disagreement? (Goodwin and Darley 2008, pp. 1362–1363)

Basically, subjects in both studies could choose between two kinds of answers: either they stated that one of the disagreeing parties is right and the other one wrong; or that it is possible or actually the case that both are right.^14^ Goodwin and Darley’s and Wainryb et al.’s interpretation of these answers was almost identical. “One of the parties of the moral disagreement is right, the other wrong” responses were taken to indicate realism. “It is possible that both are right/both are right” responses were interpreted as indicating subjectivism (see Goodwin and Darley 2008, pp. 1344–1345; Wainryb et al. 2004, p. 692):

1. One party in the disagreement is right, the other wrong = Realism
2. It is possible that both are right/both are right = Subjectivism

The main problem with Goodwin and Darley’s and Wainryb et al.’s methodology is again their inadequate metaethical assumptions. First, as they describe the moral disagreements at issue, R1 is not only entailed by realism, but also by various non-individualistic variants of subjectivism. Consider, for example, cultural relativism. According to this view, to judge a thing good means to judge that the members of the culture within which the judgement is made predominantly believe that the thing is good. Within one particular culture there can only be one predominant view about whether a thing is good. In order for cultural relativism not to entail that one party of a moral disagreement is right and the other is wrong, this disagreement must therefore take place between members of different cultures. However, neither group of researchers promoted such an interpretation. Quite the contrary! Goodwin and Darley described the disagreeing parties as subjects of their own study, which suggests that they are students of the very same university (see 2008, p. 1362). And Wainryb et al. even presented drawings which show the disagreeing parties standing face to face to each other (2004, p. 692).

As follows from the above considerations, the researchers’ interpretation of “It is possible that both are right/both are right” responses as indicating subjectivism is inappropriate as well. Non-individualistic subjectivists should not interpret Goodwin and Darley’s and Wainryb et al.’s disagreements according to this response. For example, in order for cultural relativists to be drawn to the view that the disagreeing parties are both right it would have to be the case that the parties make their judgements within different cultures, and also that each of their judgements conforms to the majority view of their respective culture. The only variant of subjectivism which is actually reflected by R2 is individual subjectivism. After all, the parties in the disagreements likely believe what they say, and according to individual subjectivism, for an individual to believe a thing to be right, wrong, good, bad, etc. already makes it true that the thing has that moral property.

In sum, Goodwin and Darley and Wainryb et al. did not measure how many of their subjects responded as realists versus subjectivists, but rather how many responded as either realists or non-individualistic subjectivists versus individual subjectivists^15^:

1. One party in the disagreement is right, the other wrong = Realism, Non-Individualistic Variants of Subjectivism
2. Both are right/it is possible that both are right = Individual Subjectivism

**Fig. 3:**
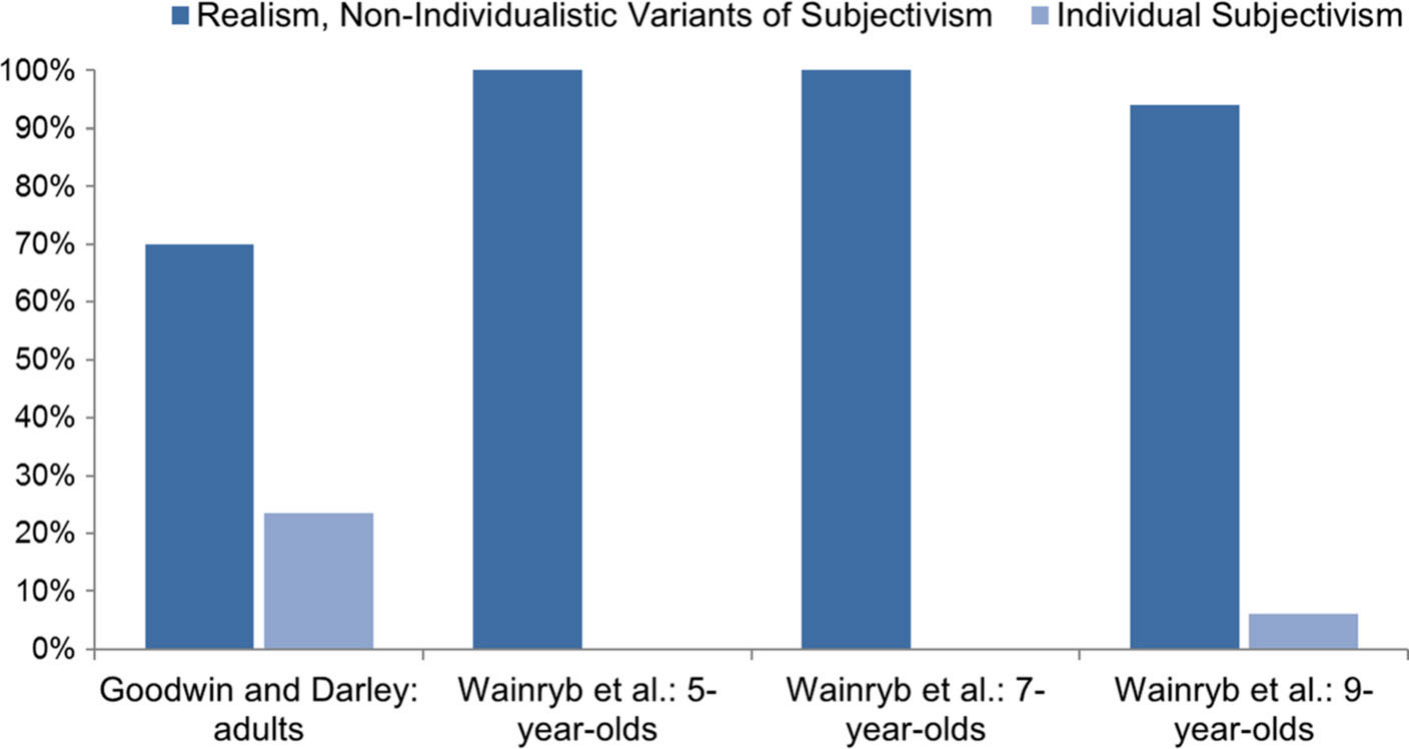
Distribution of responses in the second stage of Goodwin and Darley’s 2008 study (see pp. 1346–1350, 1352–1354, personal communication) and Wainryb et al.’s 2004 study (see pp. 693–694) as reinterpreted above

So what are the results of Goodwin and Darley’s and Wainryb et al.’s above experiments? Both groups of researchers found that subjects predominantly interpreted the moral disagreements they were presented with as involving one party that is right and one party that is wrong. In particular, it was reported that around 70 % of the responses in Goodwin and Darley’s experiments (personal communication), and 100 % of the responses of the 5-year-old, 100 % of the responses of the 7-year-old, and 94 % of the responses of the 9-year-old subjects in Wainryb et al.’s study fall into this category (2004, pp. 693–694). While these results have often been taken to support the view that ordinary people are predominantly realists (see, e.g., Goodwin and Darley 2008, p. 1359), our above reintepretations suggest that such a conclusion is in fact unsupported (see Fig. 3). All that the experiments suggest is that intuitions in favour of realism and non-individualistic variants of subjectivism are considerably more widespread than intuitions in favour of individual subjectivism — which should hardly come as a surprise, given the obvious implausibility of individual subjectivism. In fact, at least among adults the proportion of responses in favour of individual subjectivism is even higher than many metaethicists probably would have thought.

## Research Isolating Cultural Relativism

The next study on our list was conducted by Nichols (2004). Subjects in this study were presented cases in which individuals from different cultures disagree about the moral quality of an action. One of the individuals judges the action “okay”, the other individual judges it “not okay”. In Experiment 1, for example, Nichols’ vignette reads as follows:

John and Fred are members of different cultures, and they are in an argument. John says, “It’s okay to hit people just because you feel like it,” and Fred says, “No, it is not okay to hit people just because you feel like it.” John then says, “Look you are wrong. Everyone I know agrees that it’s okay to do that.” Fred responds, “Oh no, you are the one who is mistaken. Everyone I know agrees that it’s not okay to do that.” (Nichols 2004, p. 9)

Like with the studies considered in the previous Section, the question that was supposed to bring out subjects’ metaethical intuitions concerned the interpretation of these cases of moral disagreement. In experiments 1 to 4 subjects could choose from two kinds of interpretations: one according to which one of the two individuals is right and the other is wrong; and one according to which “there is no fact of the matter about unqualified moral claims” and the claim at issue is not “absolutely true or false”:

- It is okay to hit people just because you feel like it, so John is right and Fred is wrong.
- It is not okay to hit people just because you feel like it, so Fred is right and John is wrong.
- There is no fact of the matter about unqualified claims like “It’s okay to hit people just because you feel like it.” Different cultures believe different things, and it is not absolutely true or false that it’s okay to hit people just because you feel like it. (Nichols 2004, pp. 9–10)

On Nichols’ interpretation, responses of the first kind (answers 1 and 2 above) indicate realist metaethical intuitions; responses of the second kind (answer 3) indicate anti-realist intuitions (see 2004, p. 10).

1. A is right and B is wrong/B is right and A is wrong = Realism
2. There is no fact of the matter about unqualified moral claims; the moral claim at issue is not absolutely true or false = Anti-Realism

Again, however, this interpretation is metaethically inadequate. While R1 may indeed have mainly appealed to realists,^16^ the answer that there is no fact of the matter about unqualified moral claims and that the moral claim at issue is not absolutely true or false does not reflect all variants of anti-realism. Error theorists believe that there *is* a fact of the matter about unqualified moral claims: all such claims are false. Non-cognitivists believe that there is no fact of the matter about *any* moral claim, not just about unqualified moral claims. Moreover, they do not only deny that moral claims are “absolutely” true or false, but that these claims are true or false in *any* (robust) sense. The intuitions that Nichols’ second response actually mainly captures are only those of cultural relativists.

First, cultural relativism is incompatible with R1. As the disagreeing individuals are described as “members of different cultures”, and each individual’s judgement can be expected to conform to the predominant views of their respective culture (they insist that “everybody they know” agrees with them), cultural relativists are committed to the view that both individuals in the disagreements are right. Second, although R2 does not explicitly state that both individuals are right, it at least involves what may be understood as a (vague) theoretical characterization of cultural relativism. The response’s second sentence in particular (“Different cultures believe different things, and it is not absolutely true or false that it’s okay to hit people just because you feel like it”, Nichols 2004, p. 10) relates differences between the beliefs of cultures to the denial of absolute moral truths and falsities.

In sum, then, rather than the proportion of realist versus anti-realist responses, Nichols’ study seems to have mainly measured the proportion of realist versus cultural relativist responses.^17^

1. A is right and B is wrong/B is right and A is wrong = Realism
2. There is no fact of the matter about unqualified moral claims; such claims are not absolutely true or false = Cultural Relativism

**Fig. 4:**
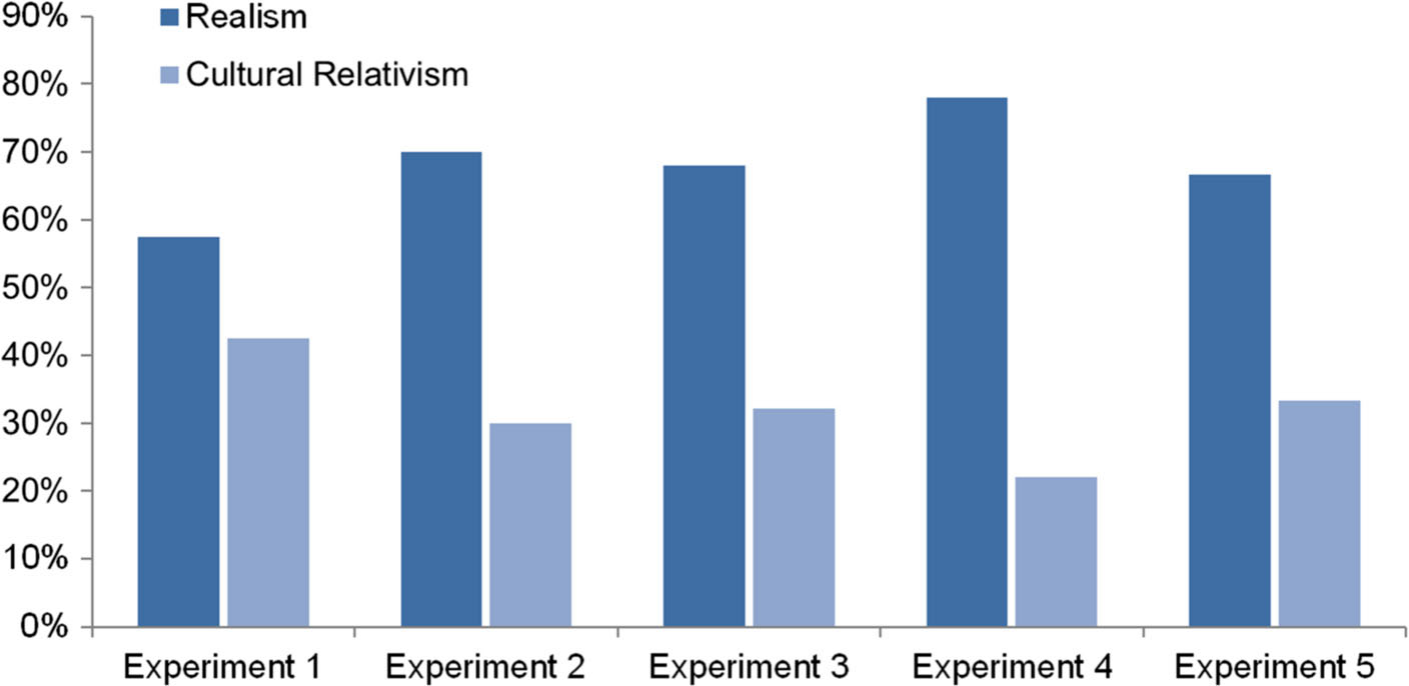
Distribution of responses in Nichols’ 2004 study (pp. 10, 16, 18–19, 20, 22) as reinterpreted above

While Nichols’ study has sometimes been claimed to show a clear tendency towards realism (e.g., Sarkissian et al. 2011, p. 484),^18^ he himself explicitly rejected such an interpretation (see 2004, p. 26). This rejection seems well-grounded, both on his original understanding of subjects’ responses and, even more so, on my above reinterpretation. Although realism was only contrasted with one particular variant of anti-realism, and although potentially confused “metaphysical relativists” (subjects who believed that ordinary physical facts are relative to the beliefs of cultures, 2004, p. 8) were excluded from analysis, realism still failed to secure strong majorities. 42.5 % of the subjects of Experiment 1, 30 % of the subjects of Experiment 2, 32.05 % of the subjects of Experiment 3^19^ and 22 % of the subjects of Experiment 4 rather preferred cultural relativism (see 2004, pp. 10, 16, 18–19, 20, 22; see Fig. 4).

Admittedly, my reinterpretation of Nichols’ results is not beyond doubt itself. At least his fifth experiment likely has sufficiently high validity, though. This experiment’s formulation of R2 reflects cultural relativism more clearly than the formulations in the other experiments. Having stressed the disagreeing individuals’ differing cultural backgrounds, Nichols puts the response as saying that there is “no objective fact, independent of what different people think” about the wrongness of the relevant actions, and that while these actions are wrong for some people, they are not “*objectively wrong* independent of what people think about them” (2004, p. 21).^20^ Moreover, the fifth experiment’s vignettes and responses also suggest more clearly that the disagreement at issue is of a specifically moral kind. They do not only speak about the relevant actions being “okay” or “not okay”, but also about their being “wrong” (2004, p. 21). So what is the result of this most trustworthy part of Nichols’ study?

As it happens, Nichols’ fifth experiment strikingly confirms the findings of Experiment 1 to 4. 33.3 % of the subjects in this experiment favored cultural relativism over realism — a figure that is almost identical to the mean of the first four experiments (31.64 %), and to the mean of all five experiments combined (31.97 %).

## Research Isolating Response Dependence Theory

The last early study on folk metaethics that I will consider in this article, conducted by Nichols and Folds-Bennett (2003), involved children between four and six years. Subjects were presented both moral sentences (e.g., “it is good for one monkey to help another hurt monkey”) and sentences about the instantiation of paradigmatic response-dependent properties (e.g., “grapes are yummy”) (2003: B26-B27). For each sentence they had to indicate whether they agreed to it. In case subjects did agree, the experimenter asked them two further questions about the sentence. The first question was designed to determine whether subjects regarded the sentence as “preference-independent”, i.e., as being true independently of the preferences of (certain) persons. In particular, subjects were asked whether they believed the sentence to be true “for some people” or “for real”.

You know, I think grapes are yummy too. Some people don’t like grapes. They don’t think grapes are yummy. Would you say that grapes are yummy for some people or that they’re yummy for real? (Nichols and Folds-Bennett 2003, p. B27)

Nichols and Folds-Bennett’s second question concerned the “generalizability” of the sentences at issue, i.e., whether these sentences are true at all times and places. To determine subjects’ attitudes about this issue they were asked whether they believed that the relevant moral or response-dependent property was instantiated “[w]ay back then, before there were any people”.

Now, think about a long time ago, before there were any people. There were still grapes, just like the grapes now. Way back then, before there were people, were grapes yummy? (Nichols and Folds-Bennett 2003, p. B27)

By comparing subjects’ answers to these questions across the moral and paradigmatically response-dependent domain Nichols and Folds-Bennett hoped to throw light on the prevalence or strength of intuitions in favor of response-dependence theory. Subjects who judged response-dependence sentences to be as preference-independent and generalizable as moral sentences were assumed to favor response-dependence theory. Subjects who judged response-dependence sentences to be more or less preference-independent and generalizable were taken to be drawn towards alternative metaethical positions (see Nichols and Folds-Bennett 2004, pp. B30-B31).

But this methodology is problematic in several respects. First, it fails to adequately measure subjects’ beliefs about the preference-independence and generalizability of the sentences at issue. Consider the question of whether a certain moral or response-dependence sentence is true “for real” or “for some people”. This question cannot only be read as asking whether the sentence is *made true* by some people accepting it, but also as another question about the sentence’s generalizability (Does it *apply* to all or only some people?) or as a purely empirical question (Is it *accepted* by all or only some people?) (Goodwin and Darley 2010, p. 167). The study’s generalizability question is no less ambiguous. Subjects may, for example, have misread it as asking whether the sentence would have been true *for them* if *they themselves* had lived “way back then, before there were any people” (Nichols and Folds-Bennett 2003, p. B28, fn. 4).^21^ Moreover, the study only addressed subjects’ beliefs about temporal, and not about spatial generalizability, i.e., generalizability across cultures.

But suppose Nichols and Folds-Bennett did succeed in determining subjects’ beliefs about the preference-independence and generalizability of moral versus response-dependence sentences. Even then their study likely would not yield any evidence about the prevalence of response-dependence theory. While their preference-independence question would only reveal whether children are drawn to a particular alternative subjectivist position, namely a position according to which moral sentences are made true by authorities or social conventions (Nichols and Folds-Bennett 2003, p. B25), the question of the generalizability of moral sentences does not have any direct implications for the study of folk moral realism at all. Neither does generalizability entail realism nor non-generalizability anti-realism — and of course, generalizability does not entail anti-realism and non-generalizability does not entail realism either (Joyce 2007b; see also Beebe and Sackris forthcoming, p. 12).

Nichols and Folds-Bennett might object that their interpretation is actually more complex. Being preference-independent and generalizable, they might argue, are necessary conditions of moral sentences. So if children believe that response-dependence sentences do not meet these conditions, they likely do not identify moral facts with response-dependent facts. However, first, that children ascribe low preference-independence and generalizability to sentences about simple response-dependent properties such as being yummy, fun, icky and boring does not mean that they would do so with regard to more sophisticated and metaethically relevant properties as well (being approved of by an ideal observer, say).^22^ And second, it is unclear whether preference-independence and generalizability are necessary conditions of moral sentences in the first place. Nichols and Folds-Bennett may take this claim to be supported by Elliot Turiel and colleagues’ finding that people generally regard moral sentences (as opposed to conventional sentences) as authority-independent and generalizable. But not only is the conceptual relevance of findings such as these contested, people may also regard a significant proportion of moral sentences as not authority-independent and generalizable at all (e.g., Kelly et al. 2007; Quintelier and Fessler 2015).^23^

In sum, the problems with Nichols and Folds-Bennett’s methodology are so grave that it cannot even be rescued by metaethical reinterpretations. Their study fails to provide any reliable evidence about the prevalence of moral realism.

## Intrapersonal Variation and Traditional Metaethics

Section 3 to 6 provided a detailed analysis of four prominent early studies on folk metaethics: studies by Goodwin and Darley (2008); Wainryb et al. (2004), Nichols (2004), and Nichols and Folds-Bennett (2003). All of these studies have been claimed to support a tendency towards realism. On closer consideration, however, we did not find any such tendency emerging. Subjects in the studies more often responded in ways that indicate non-cognitivism than cognitivism (Section 3), they at least sometimes preferred individual subjectivism to realism (Section 4), and they often favored cultural relativism over realism (Section 5).

The above findings can be explained in two distinct ways: by subjects’ intuitions having varied *interpersonally* (some were consistently drawn to one metaethical position, others consistently to other positions), and by their intuitions having varied *intrapersonally* (one and the same subject was drawn to one metaethical position with regard to some moral issues and to other metaethical positions with regard to others). Comprehensive explanations may well involve certain degrees of interpersonal variation. For the most part, however, subjects’ intuitions likely rather varied “within” them. Evidence for this interpretation comes in particular from the first stage of Goodwin and Darley’s study (Section 3). 37 of the 50 subjects of their first and 65 of the 66 subjects of their second experiment responded as cognitivists to some moral issues and as non-cognitivists to others (2008, p. 1346, 1352). For example, while many subjects considered the sentence “Robbing a bank in order to pay for an expensive holiday is a morally bad action” to be true (Experiment 1) or to admit of a correct answer as to its truth (Experiment 2), they did not concede this status to issues regarding stem cell research, abortion or assisted suicide (see 2008, pp. 1347, 1351).

Recent studies on folk metaethics have often inherited earlier research’s inadequate operational definitions of realism and anti-realism (e.g., Wright et al. 2013, 2014). Some of these studies have also been based on alternative problematic definitions (e.g., Beebe and Sackris forthcoming; Cova and Ravat 2008). To the extent that their results can be reinterpreted in reliable ways and involve more than one moral issue, however, recent studies almost unanimously suggest (and have been widely taken to suggest) very high degrees of intrapersonal variation as well. Cova and Ravat (2008), for example, found that although subjects in their study had only been presented with either two (Experiments 3 and 4) or four (Experiment 1) moral issues, almost one third of them varied in terms of their siding with realism or (what I propose to interpret as) anti-realism.^24^ Wright et al. (2013,2014) recently addressed this variation explicitly. In one of their experiments 34 out of 47 subjects gave variable metaethical groundings (2013, p. 7), and in another this was even true for all 63 participants (2014, p. 36).^25^

One possible explanation of the intrapersonal variation suggested by studies on folk moral realism is that subjects in these studies were confused. They simply did not (fully) understand what it means to say of something that it is morally right, wrong, good, bad, etc. (see Loeb 2008, p. 363). While this explanation may indeed hold true for certain subjects, Wright et al. have recently convincingly argued that at least some intrapersonal variation is rather based on a genuine competence. First, when subjects in studies on folk moral realism were asked why they had responded as they did, their verbal explanations often at least roughly reflected the metaethical positions their responses were supposed to indicate (Wright et al. 2013, pp. 349–352). And second, Wright et al. also developed a plausible theory of *why* people favour different metaethical positions on different occasions. This variation, they argue, to some extent regulates how open individuals and communities are to divergent moral judgements. The more we believe that performing some action is to be prohibited, or is not to be tolerated, the more objectivity we ascribe to the wrongness of this action (Wright et al. 2013; Wright et al. 2014).

What do a subjects’ genuinely variable intuitions mean for his/her metaethical classification? Is this subject a realist? Is s/he an anti-realist? At first sight the prevalence of intrapersonal variation may seem to have the surprising result of rehabilitating the experiential hypothesis. Many subjects in studies such as Goodwin and Darley’s, Cova and Ravat’s, and Wright et al.’s seem to have believed that while there is no objective truth about some moral issues, there is such a truth about others. These subjects thus endorsed the existence of at least some objective moral truths. And does not this suffice for considering them realists, given our definition of realism as the view that there are objective moral truths (a definition which does not refer to how *many* such truths there are or whether there are such truths with regard to *all* moral issues)? However, this attempt of rescuing the experiential hypothesis likely fails.

Traditional metaethical positions are based on two important semantic assumptions (Gill 2009, pp. 216–218; Sinnott-Armstrong 2009, pp. 237–239). According to what has been called the “determinacy assumption” (Gill 2009, p. 216), the meaning of all or at least most moral sentences is *determinate*, i.e., these sentences are either true or false in virtue of their in/correctly representing objective facts, true or false in virtue of their in/correctly representing subjective facts, or not truth-apt at all. It cannot be the case that two or more of these accounts of the meaning of moral sentences are equally correct. According to the second important assumption, the “uniformity assumption” (Gill 2009, p. 216), all moral sentences have *the same meaning*: either all of these sentences are true or false in virtue of their in/correctly representing objective facts, all of these sentences are true or false in virtue of their in/correctly representing subjective facts, or all of these sentences are not truth-apt at all.^26^

Because of realism’s commitment to the above assumptions it must be understood as entailing that *all* moral sentences *determinately* are true or false in virtue of their in/correctly representing objective facts. But subjects with intrapersonally varying metaethical intuitions obviously reject this claim. They either believe that moral sentences have both objective and non-objective meaning, or (more likely, given that their intuitions vary with the nature of moral issues and that this variation to some extent reflects a conscious strategy) that only some moral sentences have objective meaning, but others have not. In any case, these subjects reject a central assumption of realism and thus cannot qualify as favouring this position.

Of course, subjects with intrapersonally varying metaethical intuitions must not be classified as anti-realists either. Variants of anti-realism too entail that all moral sentences determinately have one and the same meaning, for example, that they determinately are all true or false in virtue of their in/correctly representing subjective facts (subjectivism) or that they determinately are all non-truth-apt (non-cognitivism). These subjects should therefore rather be regarded as *neither* realists *nor* anti-realists. They make up their own metaethical category. Following Wright et al. (2013), I suggest to call subjects with varying metaethical intuitions “metaethical pluralists” (because they endorse a plurality of metaethical positions). This means that by suggesting a very high degree of intrapersonal variation research on folk metaethics contradicts the experiential hypothesis’ claim that ordinary people experience morality as realist-seeming after all. It suggests that rather than realist-seeming, our moral experience is “pluralist-seeming”.

## Conclusion

According to the argument from moral experience, ordinary people experience morality as realist-seeming, and we have therefore prima facie reason to believe that realism is true. Some proponents of this argument have claimed that the hypothesis that ordinary people experience morality as realist-seeming is supported by psychological research on folk metaethics. While most recent research has been thought to contradict this claim, four prominent earlier studies indeed seem to suggest a tendency towards realism. In this paper I provided a detailed internal critique of these four studies. I argued that, once interpreted properly, all of them turn out in line with recent research. They suggest that most ordinary people experience morality as pluralist- rather than realist-seeming, i.e., that ordinary people have the intuition that realism is true with regard to some moral issues, but variants of anti-realism are true with regard to others.

This result is of considerable philosophical significance (see Loeb 2007b, p. 470). The broad majority of metaethicists — anti-realists as well as realists (e.g., Blackburn 2006, p. 153; Mackie 2011, p. 35) — have accepted the argument from moral experience. They have therefore operated under the assumption that in order for realists to succeed they must only defeat all plausible arguments against their view (McNaughton 1988, pp. 40–41). If the folk really do not experience morality as a realm of objective truths, however, and this experience consequently cannot possibly ground any prima facie reason for believing in the existence of such truths, then realists are challenged to provide (more) positive evidence for their view as well. And anti-realism (or pluralism) comes out as a more attractive option than has recently been thought.

## Footnotes

1 As the above references suggest, different proponents of the argument have focused on different aspects of moral experience. For a detailed discussion of these aspects see Pölzler 2014: 73–75.

2 The label “argument from moral experience” was introduced by Don Loeb (2007). One also finds the argument referred to as “presumptive argument” (Sinclair 2012), “straightforward argument” (Björnsson 2012) and “burden of proof argument” (Joyce 2009a).

3 Sometimes realism and anti-realism are defined in very different ways, for example, in exclusively semantic terms (Dummett 1978, p. 146; Sayre-McCord 1988, p. 5) or as entailing an epistemic condition (e.g., Boyd 1988, p. 182). Note that my below arguments against the experiential hypothesis may not (fully) apply on such alternative definitions.

4 By “moral” sentences discussants of realism and anti-realism mean sentences which express moral judgements, such as “Helping others is good” or “Stealing money is wrong”. Sentences *about* such sentences or judgements (i.e., metaethical or descriptively ethical sentences) do not qualify as moral.

5 This conceptual understanding of observer-independence contrasts, for example, with an ontological one (a property is objective if and only if it would be had by things if there existed no observers with mental states) and a causal one (a property is objective if and only if the fact that the property is had by things is not caused by the mental states of observers) (see Joyce 2007a; Pölzler 2014: 14–17).

6 Many contemporary proponents of non-cognitivism have argued that moral sentences are truth-apt in a non-robust (minimalist or deflationist) sense (e.g., Blackburn 2000, p. 79; Gibbard 2003, p. x). On such theories of moral truth, in saying of moral sentences that they are true we do not attribute any property to these sentences (such as the property of correctly representing the facts), but, for example, only reaffirm them.

7 My above formulation of error theory is controversial. First, error theory has often been restricted to particular kinds of moral sentences, in particular, sentences which entail that a thing has some moral property (e.g., Joyce 2007a; Pigden 2007, p. 451). And second, the relevant sentences have also sometimes been claimed to be “untrue” rather than false (Joyce 2001, pp. 6–9, 2007a). For a plausible defense of the above formulation see Loeb 2007a.

8 Response dependence theory is sometimes regarded as a variant of realism (e.g., Firth 1952, p. 322). This classification is only warranted on ontological conceptions of observer-independence (as explained in fn. 5 above), though, and not on the conceptual conception assumed here (see Joyce 2007a).

9 Notable exceptions can be found in the child development literature, where researchers have in particular explored the distinction between moral and conventional norms (e.g., Gabennesch 1990; Shantz 1982; Turiel 1978).

10 In contrast to the above mentioned psychologists, some metaethicists (e.g., Björnsson 2012, p. 369; Loeb 2008, p. 362) use the label “objectivism” to refer to positions that are distinct from realism as understood here, i.e., distinct from the view that there are objective moral truths.

11 Although Joyce endorses the experiential hypothesis and likely also some form of the argument from moral experience (see Joyce 2010), he is not a realist. His above quotation is taken from a defense of moral projectivism. As Joyce understands projectivism (Joyce 2009a, Joyce 2010), it involves two empirical claims: the claim that humans experience morality as a realm of objective facts (i.e., the experiential hypothesis), and the claim that this experience is immediately caused by affective attitudes (which he calls the “causal hypothesis”). For a critique of the causal hypothesis, based both on studies about the relation between moral judgements and emotions (Pölzler 2015) and on studies on folk moral realism, see Pölzler 2016.

12 Joyce most extensively appeals to Nichols and Folds-Bennett’s study (Joyce 2007c, p. 129).

13 Note that I will only consider those parts of the above-mentioned studies that are relevant to assessing the experiential hypothesis.

14 Goodwin and Darley also included the option “other”. Responses in favor of this option were not considered in their analysis, however (Goodwin and Darley 2008, pp. 1344, 1348).

15 That error theorist and non-cognitivist intuitions are not reflected by any of Goodwin and Darley’s and Wainryb et al.’s options is a potential problem (see Pölzler 2014: 80–81). In the case of Goodwin and Darley it is also somewhat ironic. After all, they attempted to measure the same kind of metaethical intuitions as in the first stage of their first and second experiment, which exclusively focused on cognitivism and non-cognitivism.

16 Some variants of subjectivism, in particular more sophisticated ones (such as certain forms of response dependence theory), may entail that one of the parties in Nichols’ disagreements is right and the other party is wrong as well.

17 Like in the case of Goodwin and Darley and Wainryb et al. (Section 4), Nichols’ study may be criticized on grounds of its failing to capture the full logical space of metaethical positions (see Pölzler 2014: 80–81).

18 Sarkissian et al.’s interpretation may be explained by their considering only Experiment 4 (which shows a higher proportion of realist responses than all other experiments), and by their ignoring Nichols’ exclusion of “metaphysical relativists” (see below).

19 Experiment 3 contained two realism/cultural relativism-questions. To the first of these questions, 25.6 % of subjects responded as cultural relativists, to the second 38.5 % of subjects. The figure given above represents the average proportion of subjects who responded as cultural relativists in this experiment.

20 One possible objection against my interpretation of the fifth experiment’s version of R2 is that this response is not only entailed by cultural relativism, but also by individual subjectivism. Individual subjectivists too believe that actions are wrong for you or for me, without them being objectively wrong. However, due to Nichols’ emphasis on the disagreeing individuals belonging to different cultures, his experiment likely still *mainly* plays off cultural relativist intuitions against realist ones.

21 One may object that if Nichols and Folds-Bennett had attempted to avoid all of the above ambiguities, their questions would have become too complex to be understood by children between four and six years. This consequence is indeed likely. However, rather than grounding a defense of Nichols and Folds-Bennett’s above formulations this recognition rather suggests that preference-independence and generalizability research on young children is infeasible.

22 Children between four and six may not be able to grasp more complex response-dependent concepts. But this again does not so much support Nichols and Folds-Bennett as it shows that children may not be the right kind of subjects for studies on folk metaethics.

23 For defenses of Turiel and colleagues’ findings see Sousa 2009 and Sousa et al. 2009.

24 Cova and Ravat take their non-objectivist options to indicate subjectivism. In fact, however, some of these options (believing of the disagreeing parties that “[t]hey are both wrong”, or that “[n]one of them is right or wrong”) are compatible with error theory and non-cognitivism as well. Subjects who turn out subjectivists, according to Cova and Ravat’s terminology, are thus better classified as anti-realists.

25 These experiments are not “cherry-picked”, i.e., simply those which yielded the highest proportions of intrapersonal variation, but are rather the only ones for whom Wright et al. reported these proportions. Wright et al. combined Goodwin and Darley’s measures of realism, as they were discussed in Sections 3 and 4. Properly interpreted, for a subject to have intrapersonally varying metaethical intuitions in their studies accordingly means for her/him to have been drawn to cognitivism (first stage) and realism or non-individualistic variants of subjectivism (second stage) with regard to some moral issues, and to non-cognitivism (first stage) and individual subjectivism (second stage) with regard to other issues. Note that in their 2014 study Wright et al. were not interested in the prevalence of metaethical intuitions per se, but rather in how these intuitions relate to people’s tolerance for diverging moral beliefs. For a brief summary of their respective findings and an assessment of their implications for debates about moral projectivism see Pölzler 2016.

26 Gill and Sinnott-Armstrong provide two kinds of evidence for the prevalence of the above assumptions. First, they point out that metaethicists have commonly tried to establish their semantic claims by analyzing a few examples of standard uses of moral sentences, then generalizing to these sentences as a whole (see Gill 2009, p. 217; Sinnott-Armstrong 2009, pp. 237–238). And second, they cite formulations of semantic claims that clearly reflect one or both assumptions, such as A. J. Ayer’s claim that “in every case in which one would commonly be said to be making an ethical judgment, the function of the relevant ethical word is purely ‘emotive’” (Ayer 1952: 108; note how Ayer speaks of “every case”), or Frank Jackson’s claim “By the time I have finished, you will have before you a schematic account of the meaning of ethical ascriptions and sentences in purely descriptive terms” (Jackson 1998: 113; note that Jackson does not restrict his account to only some of these ascriptions and sentences) (for these and more examples see Sinnott-Armstrong 2009, pp. 237–238).
